# Demographic and mental health profile of youth in a gender service: An African case series

**DOI:** 10.4102/sajpsychiatry.v30i0.2160

**Published:** 2024-04-17

**Authors:** Simon D. Pickstone-Taylor, Eugene L. Davids, Graham N. de Bever, Petrus J. de Vries

**Affiliations:** 1 Department of Psychiatry, Faculty of Health Sciences, University of Cape Town, Cape Town, South Africa

**Keywords:** children and adolescents, mental health, transgender, gender identity, gender nonconforming, gender-affirming, South Africa

## Abstract

**Background:**

Despite a massive global increase in research on gender-diverse youth, there have been no studies in Africa on gender-diverse children and adolescents presenting to health services.

**Aim:**

This study aimed to present the first African findings of the demographic and mental health profile of youth who have presented at a gender service in South Africa.

**Setting:**

A specialist mental health outpatient service, consisting of psychiatry, psychology and nursing input, for gender-diverse child and adolescent patients in the Western Cape.

**Methods:**

All consenting youth seen at a gender service, consisting of psychiatry, psychology and nursing input, in state and by the same clinician in private practice between January 2012 and May 2019 were participants of a retrospective, sequential case series study. Data of interest, including gender identity and sexuality, mental health history and social information, were extracted from the psychiatry files of participants.

**Results:**

Thirty-nine participants were part of the registry and qualified for the study: 72% self-identified as white, 15% as coloured and 13% as black African. The rate of co-occurring psychopathology was high (64%) and included high rates of autism, particularly in trans males (26%), suicidal ideation in 31% and a history of suicide attempt(s) in 10%.

**Conclusions:**

This first study describing gender-diverse youth seeking support relating to their gender identity in Africa showed they had remarkable similarities to those studied internationally.

**Contribution:**

Establishing that transgender youth of all major racial groups in the province with similar demographic profiles to other parts of the world are presenting to services in South Africa and in need of mental health support and interventions.

## Introduction

Over the years, there have been many debates and discussions around gender identity. Gender identity and gender roles have been considered as the behaviours, attitudes and personality traits in a particular social and historical period that are prescribed or attributed to persons of a particular gender.^1^ However, more recent references to ‘gender identity’ are concerned with how an individual perceived his or her or their gender, which may or may not correspond with the sex assigned at birth.^2^ When the gender perceived and identified with, corresponds with birth-assigned sex, the term gender-conforming or cisgender is typically used, while the opposite would be gender nonconforming. Children and adolescents who are gender nonconforming or gender-diverse may identify as transgender, genderqueer, or off the gender binary completely.^3^

Many children and adolescents who are transgender or gender-diverse form part of a vulnerable group of children and adolescents with high rates of psychopathology.^4,5^ Many of these young people often access healthcare that is not gender-affirming or might even avoid accessing it because of stigma and discrimination. Gender-affirming healthcare provides the child and adolescent with the care that is developmentally appropriate ‘orientated towards understanding and appreciating the (young person’s) gender experience’.^6^

An exploration of the landscape of studies examining gender-affirming healthcare for children and adolescents shows that most studies have been conducted in high-income settings, such as Canada,^7,8,9,10,11,12^ the Netherlands,^7^ the United Kingdom,^13,14,15^ the United States,^16,17^ Finland,^18^ Italy^19^ and Spain.^20^ Very few studies to date have presented findings from low- and middle-income settings. One study was conducted in Brazil,^21^ with a dearth in the literature on other low- and middle-income settings providing gender-affirming healthcare. To date, no studies have been published with data from the African continent in gender-diverse children and adolescents accessing healthcare, although there has been some research at the experience of gender-diverse youth attending schools in South Africa.^22^ The Professional Association for Transgender Health South Africa (PATHSA) highlighted the vulnerability of children and adolescents in the country and made clear their opposing view to a healthcare practitioner who challenged the credibility of gender-affirming healthcare for children and adolescents.^23^

Transgender and gender-diverse children and adolescents often have higher burdens of depression, anxiety, suicidal ideation and self-harm in comparison with their cisgender counterparts.^24^ The burden of psychopathology and psychiatric health is exacerbated by experiences of familial rejection, bullying, violence and discrimination.^24^ In South Africa, there are limited trained clinicians and long waiting lists for access to gender-affirming healthcare.^25^ This puts even more strain on the health and well-being of gender-diverse children and adolescents. There is also very little research on gender identity and gender diversity. To date, gender identity has not been included as an item or considered in many national surveys.^3^ This study, therefore, will be the first to describe the demographic and mental health profile of children and adolescents who have presented at a Gender Identity Development Service (GIDS) in South Africa. These findings will allow clinicians to better understand the profile and needs of transgender youth in South Africa and aid in the establishment of appropriate services for them.

## Research methods and design

The study aimed to examine the demographic and mental health profile of children and adolescents who presented at gender identity services, where the services are in line with PATHSA and World Professional Association of Transgender Health (WPATH) guidelines at a tertiary child and adolescent mental healthcare setting in Cape Town, South Africa. A retrospective, sequential case series was employed to gather the data to address the aim of the study.

### Participants

All children and adolescents seen in the GIDS between January 2012 and May 2019 were invited to provide consent for their data to be included in a research registry, after appropriate consent was collected. The GIDS was set up within the Red Cross War Memorial Children’s Hospital’s Division of Child and Adolescent Psychiatry in 2012 to evaluate and support gender-diverse youth up to the age of 18 years and their parents. Children and adolescents with concerns about their gender identity are referred to the service. Gender-diverse youth are assessed by lead author and psychiatrist with parents or carers being part of the assessment. They and their parents are offered an evaluation, understanding and support for their gender diversity. The gender-diverse youth are referred on to endocrinologists, psychologists and other healthcare providers as appropriate, along WPATH guidelines. The Red Cross Children’s Hospital is part of the public health system and is based in Cape Town. It was opened in 1956 and became the first dedicated children’s hospital established in sub-Saharan Africa. It is affiliated with the University of Cape Town and is one of two main hospitals in the Western Cape province of South Africa to provide tertiary and quaternary child healthcare. The GIDS is open to all children and adolescents in South Africa needing to access specialist support. All the children and adolescents seen at the GIDS have been evaluated by the lead author. To date, most of the children and adolescents who have accessed the GIDS have been from the Western Cape, South Africa. The registry also includes gender-diverse patients who are seen by the lead author in his private practice. These patients and families approached the GIDS but chose to be seen in the lead author’s private practice in order to access an assessment sooner and as they had private insurance or funds enabling them to do so. They received the same assessment by the lead author as those seen at the GIDS. Where a referral for hormones was appropriate, they were all referred on to the same endocrinology service in the state sector. This study does not discuss the services accessed after assessment by participants, only the demographics at assessment. Participants were seen between January 2012 and May 2019.

### Data collection methods and analysis

All participants were children and adolescents who had been seen with their parents or carers by the lead author at the GIDS or in his private practice. He had completed a gender assessment and a general psychiatric assessment and produced a written assessment report on each of these children and adolescents. This document, as well as other information reported in the young person’s medical file, was used to collect data.

For the purpose of this study, the research team developed a data extraction sheet containing all data fields of interest including gender identity and sexuality, physical and mental health history, family-related information, schooling and social information, as well as medication history and referrals. Anonymity was ensured as these data were not linked to individuals.

After all relevant approvals, medical records of patients’ records in the GIDS registry that were assessed between January 2012 and May 2019 were accessed and data were extracted.

Data were double-checked for accuracy, and any uncertainties or missing data were clarified by the lead author, who referred to the original file or spoke to the families as necessary. Extracted data were tabulated and presented using frequency and percentages for all relevant domains of interest (demographic details, sexual orientation, mental health profile).

### Ethical considerations

Permission was sought from the University of Cape Town Faculty of Health Sciences Human Ethics Committee (HREC Reference: 254/2018) and the Red Cross War Memorial Hospital (RXH:RCC 139) to access the medical records and registry of children and adolescents who accessed the GIDS. All parents or caregivers of the children and adolescents accessing the GIDS and evaluated by the lead author in private practice provided written, informed consent for their information to be stored in a registry that would be used to advance gender-affirming GIDS research and clinical services. Children and adolescents also completed assent forms, having had the process explained to them in an age-appropriate way by the lead psychiatrist. The consent to have their information be part of the database was a separate process from consent to healthcare and lack of consent had no impact on access to healthcare. Almost every child and family gave consent. The study was conducted in accordance with the Declaration of Helsinki and all ethical principles were upheld through the data collection and extraction, analysis and write-up.

## Results

### Demographic findings

As shown in Table 1, the study sample was made up of 39 children and adolescents, 30 of whom had been seen by the lead author at the GIDS at Red Cross Hospital and 9 of whom had been seen in his private practice. Their ages ranged from 4 years and 2 months to 18 years and 1 month. When examining home language, 30 participants spoke English (77%), followed by Afrikaans (*n* = 6; 15%) and isiXhosa (*n* = 3; 8%). The religious affiliations of participants showed that 21 (54%) were Christian, 9 (23%) were Atheist, 3 (8%) Agnostic, and 2 (5%) were Muslim, Jewish or unknown. Participants self-identified their ethnic groups, where 28 (72%) were white, 6 (15%) were coloured and 5 (13%) were black African. Of the participants, 15 (38%) were assigned male at birth and 24 (62%) were assigned female at birth. Of the participants, 21 (62%) identified as trans male, followed by 14 (36%) as trans female, 3 (8%) as nonbinary and 1 participant as cis male (2%). Of the 39 participants, 28 (72%) had gender expressions that matched their appearance, 9 (23%) were androgynous and 2 (5%) whose gender expression did not match their appearance. When considering participants’ names, 19 (49%) kept their birth names, while 8 (21%) had changed to a feminine name, 10 (26%) to a masculine name and 2 (4%) to a gender-neutral name. More than 59% (*n* = 23) of the participants experienced bullying, 21% (*n* = 7) in a relationship or past relationship, 8 participants had family members who identified as Lesbian, Gay, Bisexual, Transgender and others (LGBT+), while 9 participants had relatives who identified as LGBT+, 62% (*n* = 24) had family members who were supportive, close to 64% (*n* = 25) did not have access to health insurance and 54% (*n* = 21) attended a public (government) school.

**TABLE 1:** Demographic details of participants in the study (*N* = 39).

Demographic variable	*n*	%
**Developmental stage**		
Childhood	6	15
Adolescence	33	85
**Home language**		
English	30	77
Afrikaans	6	15
isiXhosa	3	8
**Religious affiliation**		
Christian	21	54
Atheist	9	23
Agnostic	3	8
Muslim	2	5
Jewish	2	5
Unknown	2	5
**Ethnic group**		
White	28	72
Coloured†	6	15
Black African	5	13
**Sex assigned at birth**		
Male	15	38
Female	24	62
Intersex	0	0
**Gender identity**		
Trans male	21	54
Trans female	14	36
Nonbinary	3	8
Cis male	1	2
**Gender expression**		
Identity matched appearance	28	72
Androgynous	9	23
Identity did not match appearance	2	5
**Names**		
Birth name	19	49
Changed to feminine name	8	21
Changed to masculine name	10	26
Changed to gender-neutral name	2	4
**Experienced bullying**		
Yes	23	59
No	15	38
Missing	1	3
**Relationship history** ‡		
Current relationship	7	21
Previous relationship	7	21
None	19	58
**Family sexuality history**		
LGBT+ family member	8	21
LGBT+ relatives	9	23
**Familial support**		
Very supportive	24	62
Somewhat supportive	8	21
One parent supportive, rest of family not	4	10
No familial support	2	5
Resistant because of religious reasons	1	2
**Health insurance**		
Comprehensive medical insurance/aid	10	26
Hospital plan	4	10
None	25	64
**School type**		
Public school	21	54
Private school	11	28
Home school	6	15
Not in school	1	3

Note: Age (range) = 4 years 2 months – 18 years 1 month.

† , Coloured is the term mixed race members of the local community use to identify themselves in South Africa and is not a disrespectful term as it is in some other parts of the world.

‡ , Only participants who were ≥13 years were asked.

### Sexual orientation

Participants’ sexual orientation by gender identity is presented in Table 2. The results suggest that of the participants who were 13 years and older, 44% (*n* = 14) identified as heterosexual (of which 8 were trans female and 6 trans male), followed by pansexual (4 trans male, 2 nonbinary and 1 trans female) with the least prevalent sexual orientation being asexual accounting for 6% of the participants (*n* = 2; 1 trans female and 1 nonbinary).

**TABLE 2:** Sexual orientation by gender identity.

Sexual orientation†	All		Trans. female	Trans. male	Nonbinary
	*n*	%			
Heterosexual	14	44	8	6	0
Pansexual	6	18	1	4	2
Gay and lesbian	5	16	1	2	0
Bisexual	5	16	1	4	0
Asexual	2	6	1	0	1

Trans., transgender.

† , Sexual orientation data collected only from participants ≥13 years of age, one participant identifying as trans male not asked about sexual orientation because of history of sexual assault.

### Mental health profile

The mental health of the 39 participants is presented in Table 3. The results show that among the transgender and/or gender nonconforming participants, 87% (*n* = 34) presented for gender dysphoria, and the most prevalent new diagnosis made by the child and adolescent psychiatrist was autism spectrum disorder (26%; *n* = 10), 64% (*n* = 25) had a psychiatric history that included one or more diagnosis, and 79% (*n* = 31) had a family psychiatric history, where major depressive disorder was the most prevalent familial psychiatric history diagnosis (*n* = 19). Of the participants presenting at the GIDS, 8% (*n* = 3) had a learning disorder and 5% (*n* = 2) an intellectual disability. Those with a history of suicidality had a suicide attempt(s) (10%; *n* = 4) and suicidal ideation without attempt(s) (31%; *n* = 12); and those with a history of self-harm either engaged with self-harm with suicidal ideation (21%; *n* = 8) or nonsuicidal self-injury (5%; *n* = 2). Of all participants, 38% (*n* = 15) used psychotropic drugs at the time of presentation, of which Selective Serotonin Reuptake Inhibitor (SSRI) (*n* = 12) was the most prevalent. Only six of the participants had a history of substance use, 5% (*n* = 2) used alcohol only, 3% (*n* = 1) used cannabis only, and 8% (*n* = 3) used both alcohol and cannabis. In terms of mental status examination of the participants at initial assessment at the GIDS, presenting as euthymic and apsychotic was the most common finding in 64% (*n* = 25).

**TABLE 3:** Mental health profile of participants in the study.

Mental health variable	*N*	%
**Presenting complaint**		
Gender dysphoria	34	87
Suicide attempt	2	5
Major depressive disorder with suicidal ideation	1	3
Second opinion	1	3
Suitability for orchidectomy	1	3
**New diagnosis by child and adolescent psychiatrist**		
Autism spectrum disorder	10	26
Major depressive disorder	2	5
Obsessive compulsive disorder	1	3
Generalised anxiety disorder	1	3
**Psychiatric history**		
One or more diagnosis	25	64
No diagnosis	14	36
**Family psychiatric history†**		
One or more diagnosis	31	79
Major depressive disorder	19	58
Anxiety	11	28
ADHD	4	10
Bipolar disorder	6	15
Alcohol use disorder	6	15
Other substance use disorder	4	10
Dementia	2	5
Suicide	3	8
Autism spectrum disorder	3	8
Psychosis	2	5
**Learning disorders**		
Yes	3	8
No	36	92
**Intellectual disability**		
Yes	2	5
No	37	95
**History of suicidality**		
Suicide attempt(s)	4	10
Suicidal ideation without attempt(s)	12	31
None	23	59
**History of self-harm**		
Self-harm with suicidal ideation	8	21
Nonsuicidal self-injury	2	5
None	29	75
**Psychotropic use at time of presentation** ‡		
Yes	15	38
SSRI	12	31
SNRI	1	3
Methylphenidate	4	10
Antipsychotic	3	8
Melatonin	1	3
**Substance use**		
Alcohol only	2	5
Cannabis only	1	3
Both alcohol and cannabis	3	8
**Mental State Examination (at assessment)§**		
Euthymic and apsychotic	25	-
Depressed and apsychotic	9	-
Anxious and apsychotic	4	-
Euthymic and psychotic	1	-

SSRI, Selective Serotonin Reuptake Inhibitor; SNRI, Serotonin and Norephinephrin Reuptake Inhibitor; ADHD, attention-deficit/hyperactivity disorder.

† , Thirty-one participants (79%) had one or more diagnosis; percentage of individual diagnoses not presented as some diagnoses are comorbid;

‡ , Eleven participants on only one psychotropic drug, percentage of individual drugs not presented as some used more than one simultaneously;

§ , Individual percentages not presented as more than one category was relevant.

## Discussion

In this study, we set out to describe, for the first time in an African context, the demographic and mental health profile of children and adolescents who presented at a gender-affirming GIDS at a tertiary child and adolescent mental healthcare unit in Cape Town, South Africa. Using a sequential case series design, 39 children and adolescents were included. The results showed that the children and adolescents who presented were largely identified as being trans male, heterosexual, with gender expression matching their identity, and had family that was supportive. Overall families had little to no access to health insurance. Study’s findings also identified that most of the participants experienced bullying and had at least one psychiatric diagnosis and a family history of a psychiatric diagnosis. Given the significant lack of any gender identity developmental data in an African context, the study provides a baseline for comparison to other African, low and/or middle-income and international findings.

### Demographic profile

Gender identity can be defined as children and adolescents’ ‘internal felt sense of self’.^26^ In this study, 90% of the children and adolescents’ gender identity was either trans male or trans female. Transgender children and adolescents form part of an underserved and underresearched population who have unique physical and mental health needs.^24,26^ This study, therefore, aimed to present an initial profile of children and adolescents who presented at a GIDS in Cape Town, South Africa, to outline the demographic and mental health profiles. The participants in the study largely experienced bullying as a result of their identification as trans- or gender-diverse; it has been reported that bullying among trans- and gender-diverse children and adolescents hampers their mental health and well-being.^27,28,29^ One of the factors that was seen as protective of mental health has been the care and support of transgender and gender-diverse children and adolescents. Close to 60% of the participants in this study perceived their parents as supportive. About 90% of parents chose to support gender-affirmative interventions along WPATH guidelines following assessment (outcomes not reported here, but further studies into outcomes would be helpful).

Many of the participants who accessed the GIDS did not have access to health insurance (medical aid); Tordoff and colleagues^27^ have highlighted that many transgender and gender-diverse young people who lack health insurance coverage are able to access state-funded healthcare services that are gender-affirming and would otherwise be seen as a barrier to care if not available. Nahata et al.^2^ found that many transgender and gender-diverse children and adolescents are limited in their access to appropriate care when having limited to no health insurance. When health insurance becomes a barrier to accessing healthcare among a marginalised group of young people who are prone to mental health challenges, self-injurious behaviour, as well as stigmatisation, and victimisation occur. Healthcare professionals need to lobby and work together with policymakers to provide equitable healthcare and advocate for health insurance reform.^2^ Although a state-funded service, the majority of young people seen came from middle class backgrounds, reflecting which populations tend to access care, rather than the demographics of the Western Cape. Sadly, it is likely that many gender-diverse youth from poorer communities never make it to the GIDS. As a powerful anecdote, the lead author gave a talk in September 2018 to mental healthcare workers providing psychological counselling and support to youth in one of the poorer townships of Cape Town and was told by two clinicians that they had known three transgender teenagers in the previous 3 years and that all of them had died by suicide.

### Sexual orientation

As with other studies internationally, this group showed a far higher level of diversity in sexual orientation than in other populations: 44% were heterosexual, 16% gay and lesbian and 34% bisexual (16%) or pansexual (18%). It is important that clinicians are mindful of this greater diversity and do not assume a certain sexual orientation. Some of this greater diversity is probably accounted for because of the high incidence of participants being on the autism spectrum, where more diversity of sexual orientation has been reported. Perhaps having had to explore their gender identity, unlike most cisgendered children, these youth have been more open to exploring and accepting more sexual orientation diversity in themselves as well.

### Mental health profile

Transgender and gender-diverse children and adolescents experience a unique health burden, particularly related to their mental health.^24,27^ The mental health of transgender and gender-diverse young people is often hampered as a result of diminished social support, heightened stigma and discrimination.^25^ More than half of the transgender and gender-diverse children and adolescents in this study had at least one psychiatric diagnosis, highlighting the unique mental health burden of these young people. Previous studies have reported poor mental health outcomes and the presence of a psychiatric diagnosis for transgender and gender-diverse young people.^27,30,31^ The mental health burden of transgender and gender-diverse children and adolescents indicates the need for more gender-affirming medical and mental health interventions to assist in the unique mental health challenges experienced by these young people.

In this study, transgender and gender-diverse children and adolescents initially accessed the GIDS because of gender dysphoria; upon further investigation and screening, many of the participants also received a new diagnosis of autism spectrum disorder. Previous studies have reported heightened autistic traits in transgender and gender-diverse young people,^32,33^ which has clinical implications for mental healthcare that is gender-affirming and adequate to the neurodiverse needs of trans- and gender-diverse young people. Additional mental health concerns seen in this study and reported by Tordoff et al.^27^ include self-harm, suicidality, anxiety and depression. These mental health concerns highlight the unique mental health burden of transgender and gender-diverse children and adolescents who are often underserved in terms of mental and physical health as well as underresearched.

### Study limitations

It is likely that there are large numbers of transgender youth who are never referred to GIDS services, whether in government or private settings, particularly in poorer and more conservative communities. This could be for many reasons including lack of funding to get to health services, lack of awareness of the service and parents who would not access such services because of their own transphobic and homophobic views. Community-based studies, for example, townships and rural communities, may provide far more representative data on rates and profile of transgender youth in South African and African contexts. While this study established that a significant sample of the transgender youth were coloured (15%) or black (13%) population, overall numbers were very low. Ongoing and larger-scale studies are therefore required to expand and ensure the representativeness of all our communities.

This study is also limited given that it was only from one South African province, the Western Cape. However, there are no other well-established gender clinics currently seeing transgender youth in Africa. There are a handful of clincians in South Africa supporting gender-diverse youth. Similar studies of the patients they are seeing or at gender clinics hopefully to be established in other parts of South Africa or Southern Africa in the future would help address this limitation.

Patients attending the GIDS filled in a standard demographic data form; however, there were no standardised assessment tools (e.g. ADOS-2 or similar) used in this study. However, each of the gender-diverse youths and families was seen by the same senior clinician who produced a detailed assessment report on each patient. The clinical diagnoses, for example, of autism spectrum were made by the clinician without a formal assessment tool, for example, Autism Diagnostic Observation Scedule-Second Edition (ADOS-2) or Diagnostic Interview for Social and Communication Disorders (DISCO). However, of the 10 patients diagnosed with autism spectrum disorder (ASD), 5 were able to afford to access a DISCO evaluation, and all 5 of them had autism spectrum confirmed. The lead author and clinician making the diagnosis is part of a clinical team that specialises in evaluating and treating youth with autism spectrum and has thus had special training and experience in this area. Further studies evaluating all patients for autism spectrum using the same standardised gold standard measures would be helpful.

The focus of this study was on risks, vulnerabilities and clinical needs, not on resilience and other positive attributes of young people. This is a limitation of this study, but an important area for future research.

## Conclusion

This study established that gender diversity is present in all the different linguistic, racial and religious groups typically seen in the Western Cape. In spite of the high degree of differences in cultural, linguistic, socioeconomic profiles and culture-sexual histories in Africa from youth in other parts of the world, this study of South African transgender youth reported similar demographics and clinical profiles seen in transgender youth seen in other parts of the world. Significantly high levels of ASD, particularly in trans males, were noticed in this sample and further studies are needed to explore this in more diverse local samples.

More education on gender diversity and appropriate referral routes is needed for clinicians working with people living in poorer communities, as well as for the members of these communities themselves. Appropriate healthcare services for transgender youth in other parts of South Africa need to be established and ways to improve access for black and Coloured youth need to be considered.
